# Passive Outdoor Host Seeking Device (POHD): Designing and Evaluation against Outdoor Biting Malaria Vectors

**DOI:** 10.1155/2020/4801068

**Published:** 2020-07-01

**Authors:** Stella T. Kessy, Ladslaus L. Mnyone, Bruno A. Nyundo, Issa N. Lyimo

**Affiliations:** ^1^Department of Environmental Health and Ecological Sciences, Ifakara Health Institute, P.O. Box 53, Off Mlabani Passage, Ifakara, Morogoro, Tanzania; ^2^College of Natural and Applied Science, Department of Zoology and Wildlife Conservation, University of Dar es Salaam, P.O. Box 35064, Dar es Salaam, Tanzania; ^3^Sokoine University of Agriculture, Pest Management Centre, P.O. Box 3110, Morogoro, Tanzania; ^4^School of Public Health, Faculty of Health Sciences, University of the Witwatersrand, Johannesburg, South Africa

## Abstract

Odor-baited devices are increasingly needed to compliment long-lasting insecticidal nets (LLINs) and indoor residual spraying (IRS) for control of residual malaria transmission. However, the odor-baited devices developed so far are bulky, dependent on the source of electricity and carbon dioxide (CO_2_), and they are logistically unsuitable for scaling up in surveillance and control of malaria vectors. We designed a passive and portable outdoor host seeking device (POHD) and preliminarily evaluated suitable components against *Anopheles arabiensis* that maintains residual malaria transmission. Experiments were conducted using semifield reared *An. arabiensis* within the semifield system at Ifakara Health Institute (IHI) in southeastern Tanzania. These mosquitoes were exposed to Suna traps® baited with BG lures or source of light and augmented with carbon dioxide (CO_2_) in view of identifying best attractants necessary to improve attractiveness of designed POHD. Two Suna traps® were hanged at the corner but outside the experimental hut in a diagonal line and rotated between four corners to control for the effect of position and wind direction on mosquito catches. Furthermore, mosquitoes were also exposed to either a bendiocarb-treated or bendiocarb-untreated POHD baited with Mbita blend, Ifakara blend, and worn socks and augmented with warmth (i.e., 1.5 liter bottle of warm water) inside an experimental hut or a screened rectangular box. This study demonstrated that mosquitoes were more strongly attracted to Suna trap® baited with BG lures and CO_2_ relative to those traps baited with a source of light and CO_2_. The POHD baited with synthetic blends attracted and killed greater proportion of *An. arabiensis* compared with POHD baited with worn socks. Efficacy of the POHD was unaffected by source of warmth, and it was reduced by about 50% when the device was tested inside a screened rectangular box relative to closed experimental hut. Overall, this study demonstrates that the POHD baited with synthetic blends (Mbita and Ifakara blends) and bendiocarb can effectively attract and kill outdoor biting malaria vector species. Such POHD baited with synthetic blends may require the source of CO_2_ to enhance attractiveness to mosquitoes. Further trials are, therefore, ongoing to evaluate attractiveness of improved design of POHD baited with slow-release formulation of synthetic blends and sustainable source of CO_2_ to malaria vectors under semifield and natural environments.

## 1. Introduction

The application of odor-baited technologies to augment the existing vector control tools against residual malaria transmission which occurs largely outdoors in most African countries started receiving noticeable attention since the 1990s [1–3]. The Long-Lasting Insecticide Treated Nets (LLINs) and Indoor Residual Spraying (IRS) are the current frontline interventions that target almost exclusively indoor biting mosquito vectors leading to increased early morning and evening, outdoor biting malaria vectors [1–3]. While the tools for surveillance and control of these malaria vectors are inefficient, they continue maintaining residual transmission of malaria. However, the current odor-baited devices are logistically impractical for mass application against outdoor biting malaria vectors in rural settings of Africa because of their unsuitable attractant delivery systems, components layout, and design.

Designing and development of odor-baited devices exploit the understanding that mosquito disease vectors locate hosts by integrating chemical, physical, and visual cues [4]. These chemical and physical cues are making a software component of a designed device. The chemical cues have an overwhelming role in dictating the attractiveness of odor-baited devices to mosquitoes relative to the other cues [5, 6]. Several volatile compounds identified from human emanations were widely studied and demonstrated attractiveness to major malaria vectors such as CO_2_, carboxylic fatty acids, oxocarboxylic acids, ketones, phenols, lactic acids, and ammonia [4, 7–12]. These volatile compounds were constituted to standardized blends used in odor-baited devices for sampling mosquitoes [9, 13–15] [10, 16]. Examples of such synthetic blends include BG lure [17], Mbita blend [16], and Ifakara blend [18]. The slow-release formulations of these synthetic lures are similarly attractive to anophelines and culicines under field settings [12, 16, 18]. Furthermore, studies have demonstrated far improved attractiveness of traps to mosquitoes when these and other blends are deployed in combination with CO_2_ [4, 10–12, 19–21] and physical cues such as heat, moisture, and light [22–25]. Since the discovery of these synthetic blends [16, 18], several prototypes of odor-baited devices were developed such as BG sentinel trap [26], Suna trap® [23], Mosquito Magnet X traps [27], Ifakara Odor-baited Station and Mosquito Landing Box [18, 28, 29], and Mosquito Trapping Box [30]. Despite promising preliminary results of these prototypes, most, if not all, are not yet ready for large-scale trials and control of outdoor malaria transmission [18, 23, 28, 29, 31, 32]. The application of current odor-baited devices for surveillance and control of malaria vectors is restricted by inefficient odor blend delivery systems [16, 18, 28–30], inefficient trapping and killing mechanisms [18, 19, 28–30, 33], nonportable physical design [18, 21, 28–30], dependence on electrical power source [10, 16, 21, 28–30, 34], and nonsustainable source of carbon dioxide [10, 16, 28–30, 34]. To make this approach logistically feasible and practical to implement in resource-poor areas, the future outdoor odor-baited devices that are portable, nondependent on the source of CO_2_, and electrical power “passive” are more desirable.

Alternatively, passive odor-baited devices are increasingly designed and developed for the sampling and controlling outdoor biting mosquitoes [21, 35–39]. The designed passive devices were tested mostly for sampling *Aedes* and *Culex* mosquitoes [36, 39], but very few of them were successfully developed against *Anopheles* mosquitoes [21, 38, 40]. Our study builds on these previous findings to design a passive outdoor host seeking device (POHD) for surveillance and control of outdoor biting malaria vectors. This designed POHD becomes affordable, portable, and nondependent on an external power source and artificial carbon dioxide (CO_2_) in order to enhance its mass application in rural settings of Africa. Therefore, our study aimed to design and develop prototypes of POHD and identify appropriate olfactory and physical cues for maximum attractiveness POHD to malaria vectors. The specific objectives of the current study were (1) to evaluate the additive/synergistic effects of CO_2_ to the attractiveness of traps baited with lures or light to malaria vectors, (2) to design attractant delivery system for POHD and compare different attractants against malaria vectors, and (3) to establish if source of heat is necessary to enhance mosquito attraction and landing reflex on POHD.

## 2. Materials and Methods

### 2.1. Study Area and Semifield System (SFS)

**Figure 1:**
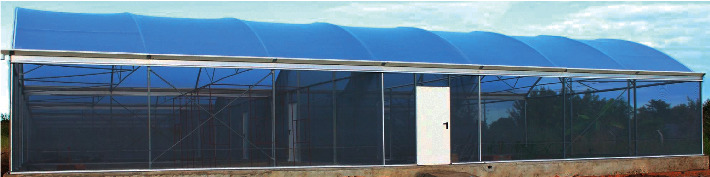
A picture of the semifield system (SFS) located at Ifakara Health Institute in Kilombero Valley, southeastern Tanzania. These chambers of SFS facility were used to conduct the evaluation of software and hardware components of designed POHD.

All experiments in this study were conducted within the semifield system (SFS) at Ifakara Health Institute (IHI) in Kilombero Valley, southeastern Tanzania (Figure 1). These experiments were replicated in several independent chambers with a dimension of 2.97 × 6.70 × 2.80 m within the SFS. The main malaria vectors which are dominating residual malaria transmission within this valley include *An. arabiensis* and *An. Funestus*, but *Anopheles gambiae s.s* has declined to undetectable levels [41–45]. Over 95% of the malaria vector population in this valley is composed of *An. arabiensis* [46].

### 2.2. Semifield Reared Mosquitoes

The wild population of *An. arabiensis* was a founder of semifield reared *An. arabiensis* for these experiments [47, 48]. The semifield reared colony of *Anopheles arabiensis* mosquitoes used in this study was established in 2008 by collecting blood-fed individuals from Sagamaganga village in Kilombero district (8.0667°S, 36.8000°E). The vector species composition in this valley is composed of over 95% of *An. arabiensis* [46]. These mosquitoes were reared in a chamber of 9.1 × 9.6 × 3.7 m within the SFS following procedures described elsewhere [47, 48]. The ambient temperature and relative humidity in that chamber ranged from 25°C to 32°C and from 70% to 90%, respectively, and they were almost similar to the natural environment [47–49]. Our previous work demonstrated that ecological diversity influences the population genetic structure of wild *An. arabiensis* [50]. When these mosquitoes were reared within semifield systems as the free-flying population as in natural environments, they retained genetic variability, inbreeding rates, lipids, and body size similar to their founding wild populations, but they slightly lost these traits under small cages colonization after at least 10 generations [49, 51]. The semifield reared adult mosquitoes were maintained with a 10% glucose solution and human blood for the propagation of the population. For experiments, the female mosquitoes of 3–7 days old from stock cages were starved for six hours before experimentation.

### 2.3. Standard Attractants and Killing Agents for POHD

**Figure 2:**
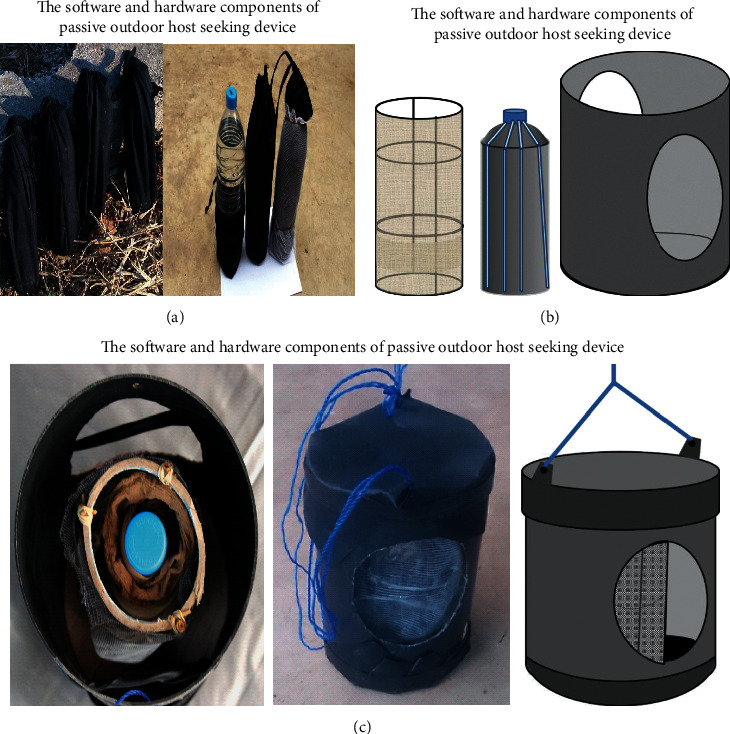
A prototype of a passive outdoor host seeking device (POHD). The designed POHD is made up of software and hardware components. The software components included (a) a bottle of water inside black cotton sack kept under solar power and (b) a bottle of water with nylon strips of attractants and a metal frame surrounded with electrostatic bendiocarb-treated or bendiocarb-untreated netting. The hardware component includes (c) the polyvinyl chloride (PVC) outer cover with two holes on sides, covered on top or bottom to protect the software components of the device.

These experiments tested standard attractants and mosquito killing agents (bioactive) as software components of designed POHD to attract and kill mosquitoes, respectively (Figure 2). Therefore, this study evaluated different olfactory and physical cues to identify suitable attractants for the designed POHD (Figure 2). The sources of olfactory cues were the Ifakara blend [18], Mbita blend [16], BG lure [17], the yeast-produced CO_2_ [10, 52, 53], and human worn socks [19]. The socks were worn by a male volunteer of 24 years old for 12 hours to collect human skin odors as a source of attractants. The fermented produced CO_2_ was generated from a mixture of fermented yeast and sugar solution as described elsewhere [10, 52, 53]. Another software of designed POHD was the physical attractants such as heat and light sources. While the light was generated from an electric bulb, the heat source applied inside POHD was a 1.5-liter bottle of warm water wrapped inside black cloth sack and heated up under the sun for approximately 12 hours (Figure 2(a)). The last software component of POHD was the wettable powder formulation of bendiocarb applied on a piece of SAFI netting or electrostatic netting [54]; it was fixed around the source of lures to kill any single mosquito that visited the POHD as a proxy for determining the proportion of attracted mosquitoes.

### 2.4. Experimental Procedures

#### 2.4.1. Assembling the Software and Hardware Components of Designed POHD

The designed POHD was composed of inner software and outer hardware components (Figure 2). The inner software components were as follows: 1.5-liter plastic bottle with warm water wrapped inside cotton cloth sack as the source of the physical cue, the synthetic nylon strips of attractive blends (i.e., Mbita blend and Ifakara blend), and worn nylon socks as olfactory cues; and the bendiocarb-treated electrostatic netting around the metal frame (Figures 2(a) and 2(b)). The outer hardware component of POHD is a plastic cover of polyvinyl chloride (PVC) of 0.16 m diameter × 0.47 m height for protection of software components (attractants and bendiocarb powder) and enhancing mosquito trapping mechanism of designed POHD (Figure 2(c)).

#### 2.4.2. Assessing the Synergistic Role of CO_2_ in Odor-Baited Mosquito Traps

**Figure 3:**
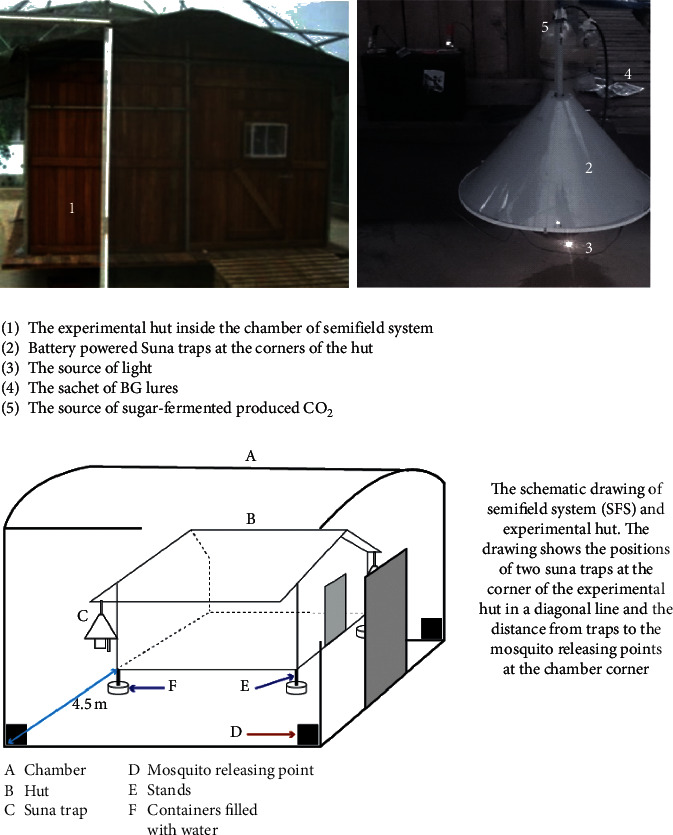
Experimental system used to assess synergistic and/or additive effect of carbon dioxide on the attractiveness of BG lures and light baited Suna traps to *Anopheles arabiensis*. The experimental hut was placed at the center of SFS chamber (1), Suna trap (2) hanged at the corner of the hut, Suna trap baited with a light source (3), Sachet of BG lures (4), and the source of CO_2_ generated from the yeast fermentation of sugar (5). The schematic drawing of the experimental system shows the position of Suna traps hanged at the corner of the hut and the distance from the traps to the mosquito releasing points at the corner of the chamber of SFS.

The experiments to preselect the suitable combinations of chemical and physical cues to be integrated inside the designed POHD were conducted using standardized Suna traps® inside the chamber (9.1 × 9.6 × 3.7 m) of the SFS (Figure 3). The BG lures tested were lactic acid (LA) and ammonia (NH) leading to a combination of treatments as follows: lactic acid alone (LA), lactic acid and CO_2_ (LA + CO_2_), ammonia alone (NH), ammonia and CO_2_ (NH + CO_2_), ammonia and lactic acid (NH + LC), and ammonia, lactic acid, and CO_2_ (NH + LC + CO_2_). Additionally, Suna traps were also baited with a physical cue of light alone (LT) or combination of light and CO_2_ (LT + CO_2_) to test if POHD would require a combination of chemical and physical cues to enhance its attractiveness to mosquitoes (Figure 3). These baited Suna traps were hanged at the corner, outside an experimental hut (3.5 × 4 × 2.5 m), which was constructed and placed at the center of the chamber of SFS (Figure 3). Each night of the experiment, two different baited Suna traps were randomly hanged at 25 cm from the ground in a diagonal line of two different corners of the experimental hut (Figure 3). A total of 200 female *An. arabiensis* starved for 6 hours were released at 6 : 30pm outside of experimental hut in batches of 50 mosquitoes per corner of the chamber and left to forage overnight. The mosquito releasing point was set at 4.5 m equidistant from each corner of the hut where the source of attractants is placed to the corner of the chamber of SFS (Figure 3). Furthermore, the Suna baited traps were rotated across four corners of the hut to minimize the positional and wind direction effects on the variations in mosquito catches. The next day, mosquitoes were collected from inside Suna traps and chamber of the SFS, counted, and recorded. All these treatment combinations were tested in three replicates (Table 1).

#### 2.4.3. Assessing Attractiveness of Designed POHD to Mosquitoes

**Figure 4:**
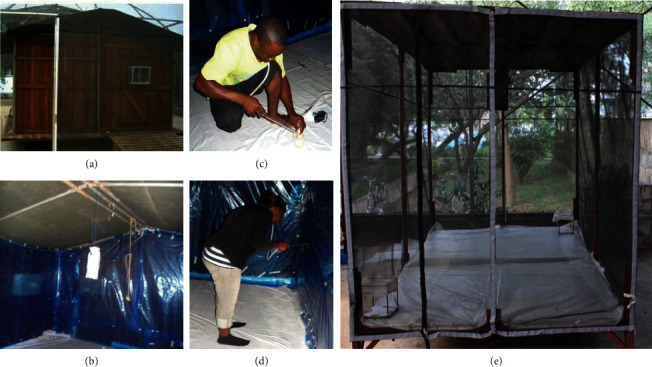
Experimental systems used to evaluate the attractiveness of a passive outdoor host seeking device against *An. arabiensis*. Experiments were conducted under closed (air-tight) environment using (a) the experimental hut within the SFS and (b) bottle of water surrounded with attractants and treated or untreated netting hanged in the middle of the experimental hut. (c-d) The alive and dead mosquitoes were collected from walls, floors, and roofs of the hut. Other experiments were conducted under free wind movement inside the screened rectangular bioassay box with a complete device hanged in the middle (e).

The experiments to evaluate the attractiveness of designed POHD to mosquitoes were conducted inside an experimental hut with dimensions of 3.5 4 × 2.5 m (Figures 4(a)–4(d)). These experiments tested the attraction and landing responses of mosquitoes to the designed POHD. The designed POHD had the following components: the warm bottle as a source of physical cues (warmth), nylon strips of synthetic blends (i.e., Mbita blend) and worn socks as chemical cues, and bendiocarb-treated netting around the metal frame as mosquito killing agent. The following treatment combinations in POHD were exposed to mosquitoes: (i) warmth + Mbita blend + untreated netting (control device) and (ii) warmth + Mbita blend + bendiocarb-treated netting (treated device). The warmth was generated by a bottle of water packed inside the black cotton sack and exposed under the sun for 12 hours during the day (Figure 2). In the evening of the experimental night, the bottle with warm water was covered by nylon strips of the Mbita blend followed by a rectangular piece of bendiocarb-treated netting (Figure 2). The piece of SAFI netting was treated by dipping in 80% bendiocarb solution (0.137 g bendiocarb in 11 ml of water) (Figure 2). The designed POHD baited with appropriate components was suspended at the center of the air-tight (sealed) experimental hut (Figure 4(b)). In the evening (i.e., 7 : 00 pm), a total of 200 female *An. arabiensis* were released at four different corners inside the hut, 50 mosquitoes per each corner, and left to forage for overnight (Table 2). In the next morning (i.e., from 6 : 00 am), all alive mosquitoes inside the experimental hut were collected by mouth aspirator, counted, and recorded (Figures 4(c) and 4(d) and Table 2). The dead mosquitoes on the floor of the experimental hut were also collected, counted, and recorded (Figures 4(c) and 4(d) and Table 2), but the alive mosquitoes were held in the semifield insectary and monitored for 24-hour mortality rates. The proportion of dead mosquitoes was taken as the total mosquitoes found dead on the floor of a hut in the morning (immediate dead) and those which died after 24 hour-delayed mortality rates (Table 2). All experiments inside the experimental hut were replicated three times.

The next experiment on the attractiveness of designed POHD to mosquitoes was also conducted inside a screened rectangular box with a dimension of 2.06 × 1.50 × 1.47 m (Figure 4(e)). The format of POHD tested inside the screened rectangular box was slightly modified by including an outer PCV cover with holes on sides and cover on top and bottom of the device (Figure 2), and two different synthetic blends (Ifakara or Mbita blends) were separately added in a designed POHD. The following treatment combinations were evaluated under this setup: (i) warmth + Mbita or Ifakara blend or worn socks + untreated netting (control device) and (ii) warmth + Mbita or Ifakara blend or worn socks + bendiocarb-treated netting (treated device). The preparation of other components of the POHD was done the same way as in the experiment inside the experimental hut (Figures 2(a) –2(c)). Under this setup, the bendiocarb powder applied on electrostatic netting was used unlike in the previous experiment above where a wettable solution was applied on SAFI netting. The designed POHD baited with treatments above was assembled and suspended inside the screened rectangular box (Figure 4(e)). A total of 100 mosquitoes were released inside the screened rectangular box at its entrance and left to forage overnight (Table 2). The next morning, all alive mosquitoes inside the screened rectangular box and the POHD were collected by mouth aspirator from screen and floor, counted, and recorded (Figure 4(e) and Table 2). The dead mosquitoes on the floor of the screened rectangular box were also collected, counted, and recorded (Figure 4(e) and Table 2), but the alive mosquitoes were held in the semifield insectary and monitored for 24-hour mortality. Therefore, the proportion of dead mosquitoes was derived as the total mosquito found dead on the floor of a box (immediate dead) and those mosquitoes found dead after 24 hours (Table 2). All experiments inside the screened rectangular box were replicated three times.

#### 2.4.4. Assessing the Synergistic Effect of Warmth to Baited POHD

These experiments were conducted inside a screened rectangular box to establish if the passive source of heat (warmth) inside the designed POHD baited with the Ifakara blend can enhance mosquito attraction and landing responses on the device more than those traps without warmth. As in the experiments above, the 1.5-liter bottles with water were exposed under the sun for 12 hours, and it was placed inside POHD to passively generate heat (warmth) in order to improve the attractiveness and landing responses of mosquitoes to POHD baited with the Ifakara blend. The device was also treated with bendiocarb-electrostatic netting around the source of lures to kill any attracted and landing mosquito. The proportion of mosquitoes attracted to POHD baited with the Ifakara blend was compared between devices with and without heat source (warmth). Additionally, the temperature inside and around the bottle of warm water was also recorded using data loggers over time during the day and night to determine the warmth retention capacity of the designed POHD.

### 2.5. Ethical Considerations

Ethical clearance was obtained from the Institutional Ethics Review Board (IRB) of the Ifakara Health Institute (Ref : IHI/IRB/No.14–2013) and the Medical Research Coordinating Committee of the National Institute for Medical Research in Tanzania (Ref : NIMR/HQ/R.8a/Vol.IX/1784). Also, the National Institute for Medical Research granted permission to publish this work (NIMR/HQ/P.12/Vol.XXVIII/77).

### 2.6. Statistical Analysis

The response variables measured in these experiments were the mean mosquito catches in Suna trap®, the proportion of dead mosquitoes in designed POHD baited with synthetic blends and augmented with warmth, and temperature recorded from inside and outside components of designed POHD.

The continuous response variable of mosquito catches (count data) in Suna trap® was analyzed using generalized linear mixed models with Poisson distribution models (log link function) in the R software package [55]. The poisson distribution model was then checked for overdispersion. The overdispersion was detected by comparing the residual deviance and the residual degrees of freedom in the Poisson distribution model. If the ratio of residual deviance to residual degrees of freedom was considerably greater than 1, this indicates the evidence of overdispersion. Alternative negative binomial models were then developed and compared to the Poisson distribution models using the Akaike Information Criterion (AIC). A model with a lower AIC was chosen as the best model. Such best model was then used to generate estimates of the main effects that were then exponentiated to obtain the mean number of mosquito catches in the Suna traps. The post hoc multiple comparisons were also conducted using Tukey's test to identify the significant variations on the mean mosquito catches between different treatment combinations.

The proportion of dead mosquitoes (binomial response variable) was analyzed using generalized linear mixed effect models with binomial errors (logit link function) in the **R** statistical software package [55, 56]. Treatments and experimental conditions were included as main effects (fixed effects). The day of the experiments was included as a random effect. A base minimal model including only the random effect “day” was constructed. The main effects and their interaction term were sequentially added to a base model to create a full model. Statistical significance of fixed effects and interaction term was generated and evaluated using likelihood ratio tests (LRTs). Then, the full model was used to perform a two-way multiple comparisons using Tukey's post hoc tests (adjusting for multiple comparisons) to establish statistical differences between treatment combinations.

The continuous response variables of temperature values recorded outside and inside the heat source (i.e., bottle with warm water) of POHD were analyzed using generalized linear mixed models in **R** statistical software [55]. The “time” after every 1 hour and the position point where the temperature was recorded were considered as the main effects, but different days of recording temperature were taken as the random effects. The analysis was then conducted as in the proportion data above.

## 3. Results

### 3.1. Synergistic/Additive Effects of CO_2_ to Baited Suna Traps®

**Figure 5:**
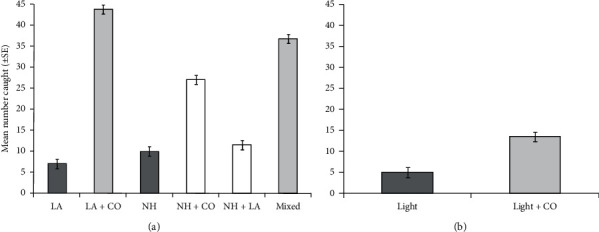
Mean catches (±1SE) of *An. arabiensis* in Suna traps baited. The Suna traps baited with (a) BG lures alone or in combination with a source of carbon dioxide (CO_2_) and (b) the source of light in combination with or without a source of CO_2_. The treatment combinations were abbreviated as follows: LA: lactic acid alone; LA + CO_2_: a combination of lactic acid and CO_2_; NH: ammonia alone; NH + CO_2_: a combination of ammonia and CO_2_; NH + LA: a combination of ammonia and lactic acid; NH + LC + CO_2_: a combination of ammonia, lactic acid, and CO_2_; LT: light alone; LT + CO_2_: a combination of sources of light and CO_2_. The grey boxes indicate statistically different comparisons.

A total of 14,400 mosquitoes were released and exposed to Suna trap® baited with olfactory cues including LA, NH, and CO_2_, but a total of 4,800 mosquitoes were exposed to Suna traps baited with physical cues of light alone or light combined with CO_2_ (Table 1). Consequently, a total of 12,062 mosquitoes were recaptured from inside the trap and around the chamber when Suna traps baited with olfactory cues (LA, NH, and CO_2_), out of which, a total of 1,666 mosquitoes (14%) were collected inside the traps. Mean mosquito catches were 4.7%, 30%, 6%, 16%, 6.4%, and 20% in the trap baited with LA alone, LA + CO_2_, NH alone, NH + CO_2_, NH + LA, and NH + LA + CO_2_, respectively (Figure 5(a) and Tables 1 and 2). In contrast, a total of 3,720 mosquitoes were recaptured from Suna traps baited with physical cues of light or combination of light and CO_2_ (Figure 5(b), Tables 1 and 2). Out of these mosquitoes, a total of 222 mosquitoes (5.9%) were collected from inside Suna traps (Tables 1 and 2). The mean catches of mosquitoes were 3.2% for traps baited with light alone and 8.7% for those baited with a combination of light and CO_2_ (Figure 5(b) and Tables 1 and 2).

The proportion of attracted mosquitoes varied significantly between Suna traps baited with individual BG lures and their different combination treatments (*χ*_5_^2^ = 662.56, *P* < 0.001, Figure 5(a)). The Suna traps baited with lactic acid (LA) alone caught a similar number of mosquitoes as those baited with ammonia (NH) alone (*z* = 2.49, *P*=0.12, Figure 5(a) and Tables 1 and 2). The source of CO_2_ had additive/synergistic effects to Suna traps baited with these BG lures. When LA baited Suna traps were augmented with a source of CO_2_, mosquito catches were significantly greater than those traps baited with LA alone (*z* = 15.77, *P* < 0.001, Figure 5(a) and Tables 1 and 3), NH alone (*z* = −14.77, *P* < 0.001, Figure 5(a) and Tables 1 and 2), combination of NH with CO_2_ (*z* = −6.90, *P* < 0.001, Figure 5(a) and Tables 1 and 2), and combination of NH with LA (*z* = −14.13, *P* < 0.001, Figure 5(a) and Tables 1 and 3), but they were similar to those traps baited with combinations of NH, LA, and CO_2_ (*z* = −2.74, *P*=0.06, Figure 5(a) and Tables 1 and 2). In comparison with LA baited traps, mosquito catches in NH baited Suna traps augmented with CO_2_ were also significantly greater than those in traps baited with LA alone (*z* = 11.15, *P* < 0.001, Figure 5(a) and Tables 1 and 2), NH alone (*z* = 9.44, *P* < 0.001, Figure 5(a) and Tables 1 and 3), and combination of NH with LA (*z* = −8.49, *P* < 0.001, Figure 5(a) and Tables 1 and 2), but they were fewer than those in traps baited with a combination of NH + LA + CO_2_ (*z* = 4.247, *P* < 0.001, Figure 5(a) and Tables 1 and 2). Furthermore, the Suna traps baited with combined NH and LA and augmented with CO_2_ had greater mosquito catches than those traps baited with NH alone (*z* = 12.804, *P* < 0.001, Figure 5(a) and Tables 1 and 2). Surprisingly, mosquito catches in Suna traps baited with a combination of NH and LA were significantly greater than those of traps baited with LA alone (*z* = 3.62, *P* < 0.001, Figure 5(a) and Tables 1 and 2), but they were similar to those in traps baited with NH alone (*z* = 1.17, *P*=0.84, Figure 5(a) and Tables 1 and 2). Similar to BG baited traps, mosquito catches in Suna trap baited with light source varied significantly between presence and absence of carbon dioxide treatments (*χ*_1_^2^ = 48.671, *P* < 0.001, Figure 5(b) and Tables 1 and 2). The Suna trap baited with a combination of light and CO_2_ had greater mosquito catches than in those traps baited with light alone (*z* = 6.57, *P* < 0.001, Figure 5(b) and Tables 1 and 2).

### 3.2. Attractiveness of Odor-Baited POHD

**Figure 6:**
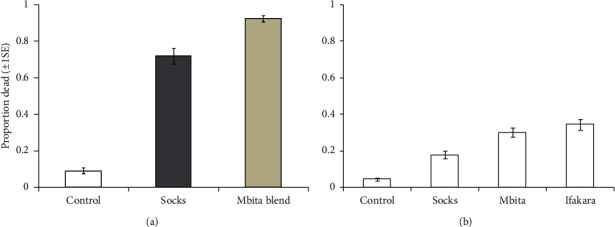
The attractiveness of designed passive outdoor host seeking device against the population of *An. arabiensis.* Estimated proportions (±1SE) of mosquitoes attracted and killed by POHD placed inside (a) experimental hut and (b) a rectangular screened box. The grey colored box indicates a statistically significant difference greater than other treatments. The error bars represent ±1 standard error.

A total of 2,400 and 1,200 mosquitoes were, respectively, exposed to odor-baited POHD inside the sealed experimental hut and screened rectangular box (Tables 3(a) and 3(b)). In the sealed experimental hut, 1,816 mosquitoes that were recaptured with 854 (55%) were dead, and 962 (45%) mosquitoes were alive (Table 3(a)). The mean proportion of dead mosquitoes was 73% in the treated device and 1% in the control device when baited with worn socks (Figure 6(a) and Table 3(a)). When designed POHD was baited with Mbita blend, the mean proportion of dead mosquitoes was 93% in the treated device and 3% in the control device (Figure 6(a) and Table 3(a)). In the screened rectangular box, a total of 1,149 mosquitoes were recovered (Table 3(b)). Out of these mosquitoes, a total of 162 mosquitoes were dead, and 1087 mosquitoes were alive (Table 3(b)). The mean proportion of dead mosquitoes was 18% for treated device and 3% for control device when baited with socks, while the mean proportion of dead mosquitoes was 30% in the treated device and 5.5% in control device when POHD baited with Mbita blend (Figure 6(b) and Table 3(b)).

The proportion of mosquitoes killed by designed POHD varied significantly between different treatments under sealed experimental hut (*χ*_2_^2^ = 813.3, *P* < 0.001, Figure 6(a) and Tables 3(a) and 4(a)). The proportion of dead mosquitoes collected from designed POHDs without bendiocarb was significantly lower than those collected from POHDs with bendiocarb both when baited with Mbita blend (*z* = −20.946, *P* < 0.001, Figure 6(a) and Tables 3(a) and 4(a)) and worn nylon socks (*z* = 17.575, *P* < 0.001, Figure 6(a) and Tables 3(a) and 4(a)). However, the designed POHDs baited with the Mbita blend had consistently attracted and killed a significantly greater proportion of mosquitoes than POHDs baited with worn socks (*z* = −7.744, *P* < 0.001Figure 6(a) and Tables 3(a) and 4(a)).

Similarly, the proportion of mosquitoes attracted and killed by the designed POHDS varied between treatments when exposed in a screened rectangular box (*χ*_3_^2^ = 209.71, *P* < 0.001, Figure 6(b) and Tables 3(b) and 4(b)). Multiple comparisons revealed that untreated POHDS attracted and killed a fewer number of mosquitoes than the POHD baited with the Ifakara blend (*z* = 11.720, *P* < 0.001, Figure 6(b) and Tables 3(b) and 4(b)) and Mbita blend (*z* = 10.684, *P* < 0.001, Figure 6(b) and Tables 3(b) and 4(b)) and from worn socks (*z* = 6.817, *P* < 0.001, Figure 6(b) and Tables 3(b) and 4(b)). Likewise, the POHDS baited with worn socks attracted and killed lower proportion of mosquitoes than the POHDS baited with the Mbita blend (*z* = −3.407, *P*=0.0035, Figure 6(b) and Tables 3(b) and 4(b)) and Ifakara blend (*z* = −4.421, *P* < 0.001, Figure 6(b) and Tables 3(b) and 4(b)). However, the proportion of dead mosquitoes was similar between POHD treated with Ifakara and Mbita (*z* = −1.097, *P*=0.689, Figure 6(b) and Tables 3(b) and 4(b)).

### 3.3. The Synergistic Effects of Warmth to Odor-Baited POHD

A total of 600 mosquitoes were released and exposed to both POHD with and without warmth as physical cues for mosquito attraction. In the experiments using POHD treated with warmth, a total of 570 mosquitoes were recaptured in which 460 (80.70%) were alive and 110 were dead (19.29%). The mean proportion of dead mosquitoes was 0.44 (44%) for a treated POHD and 0.05 (5%) for a control POHD when baited with the Ifakara blend. In the experiments using POHD without warmth, a total of 477 mosquitoes were recaptured from the box, and 357 (74.84%) mosquitoes were alive and 120 (25.16%) were dead. The mean proportion of dead mosquitoes was 0.43 (43%) for a treated POHD and 0.1 (10%) for a control POHD when baited with the Ifakara blend. Moreover, the temperature of the designed POHD varied with time both inside and outside surroundings. Inside the device, the temperature ranged from 23°C to 38°C with the mean temperature of 31°C in the day time (6 : 00 to 18 : 00) and 27°C during night time (19 : 00 to 5 : 00). Contrastingly, the temperature ranged from 22°C to 40°C outside the POHD with the mean temperature of 24°C on the day time (6 : 00 to 18 : 00) and 29.5°C during night time (19 : 00 to 5 : 00). Consequently, the designed POHD had consistently generated temperature by a range of 1.8°C to 8.7°C more than the surrounding environment, but it cooled down even before the exposure to mosquitoes from 16 : 00 pm to 19 : 00 pm.

**Figure 7:**
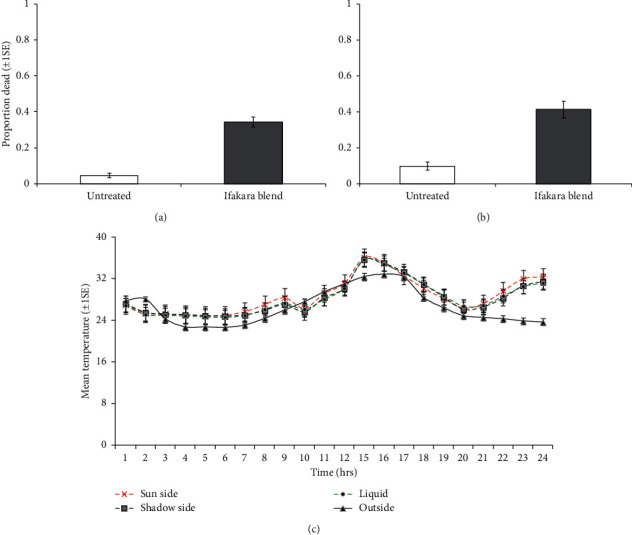
The synergistic effect of warmth to the attractiveness of a passive host seeking device against the population of *An. arabiensis*. Estimated proportions (±1SE) of *An. arabiensis* that were attracted and killed by a passive device when odors were augmented: (a) with warmth (presence of warmth) and (b) without warmth (absence of warmth). The black boxes indicate a statistically significant difference greater than the open box. Estimated mean (±1SE) of temperature recorded after every 1 hour to show variations in warmth between different environments either inside the passive device on sun side (red line), shadow side (black line, rectangular marker), in water (green dotted line), and outside the device (black dashed line, rectangular marker) within 24 hrs (warmth in POHD) (c).

The attractiveness of designed POHD against the population of *Anopheles arabiensis* was not influenced by the presence or absence of warmth (Treatment *∗* warmth status: *χ*_1_^2^ = 1.78, *P* = 0.18, Figures 7(a), and 7(b)). However, the attractiveness of POHD baited with the Ifakara blend was consistently greater than that of nonbaited POHD regardless of the presence/absence of a source of warmth (*χ*_1_^2^ = 90.23, *P* < 0.001, Figures 7(a) and 7(b)). The temperature generated by the warmth source inside POHD varied significantly across time for each position (outside POHD: *χ*_21_^2^ = 324.24, *P* < 0.001, water: *χ*_1_^2^ = 90.23, *P* < 0.001, sun side: *χ*_23_^2^ = 438.58, *P* < 0.001, shadow side: *χ*_1_^2^ = 90.23, *P* < 0.001, Figure 7(c)). In all environments (inside and outside) of POHD, the temperature recorded was highest from 12 : 00 pm to 18 : 00 pm when warming the bottle of water under solar power than during exposure of the bottle to mosquitoes from 19 : 00 pm to 6 : 00 am (*P* < 0.001, in all cases, Figure 7(c)). While heating up the bottle of water, the temperature recorded outside the designed POHD ranged from 30.96 ± 0.65°C at 12 : 00 pm to 32.86 ± 0.65°C at 16 : 00 pm, but it ranged from 30.16 ± 1.48°C at 12 : 00 pm to 36.12 ± 1.64°C at 15 : 00 pm inside the designed POHD (Figure 7(c)).

## 4. Discussion

The broad objective of this study was to design a passive odor-baited outdoor device (POHD) for surveillance and control of outdoor biting malaria vectors such as *Anopheles arabiensis*. The designing and evaluation of POHD were preceded by evaluations of different chemical cues (individually and combined) in order to select the most attractive set of cues and formats for incorporation into the designed POHD. This study tested the synergistic or additive effects of CO_2_ to the synthetic lures such as BG lures using standardized Suna traps®. The BG lures used in this study were standardized individual volatile compounds such as ammonia and lactic acids developed into slow-release (granular) formulations. These BG lures are commonly applied in odor-baited traps for sampling *Aedes* mosquitoes [12, 17], but they were also tested against malaria vectors [57]. The present study demonstrated that Suna traps baited with BG lures in combination with CO_2_ attracted *An. arabiensis* by 63.83–83.96% more than BG lures alone. This suggests that CO_2_ synergized BG lures (individually or when combined) and enhanced the attractiveness of the Suna trap to mosquitoes. However, Suna traps baited with ammonia showed mild attraction of mosquitoes when applied alone or in combination with lactic acid or CO_2_. These findings are consistent with the previous studies that reported a lack of additive or synergistic effects when traps were baited with ammonia in combination with lactic acid or CO_2_ [8, 14, 19, 57]. The traps baited with combined lactic acid and CO_2_ showed greater attractiveness to mosquitoes than other cues as lactic acid is one of the main cues present in human sweat [9, 21, 57]. Similarly, the traps baited with either specific odors or synthetic blends (e.g., Mbita blend, Ifakara blend, and BG lures) attracted more mosquitoes when augmented with CO_2_ than traps baited with blend alone [4, 5, 10–12, 19, 21, 23, 57]. Like BG lures, Mbita and Ifakara blends augmented with CO_2_ were demonstrated to be equally attractive to *Anopheles*, *Culex*, and *Aedes* mosquitoes [12, 57]. As such, a similar slow-release formulation that effectively delivered BG lures to *Anopheles* and *Aedes* mosquitoes could also be deployed to dispense Mbita and Ifakara blends to *An. arabiensis* in the field through the proposed POHD and other devices.

The initial evaluations in this study also involved experiments on additive/synergistic effects of CO_2_ to the attractiveness of light baited Suna traps® to mosquitoes to establish if designed POHD requires to integrate both physical and chemical cues. A light source in traps increases the visibility and attractiveness of the hosts or objects such as the traps to mosquitoes [36, 58]. However, the light traps placed besides human-occupied bed net (i.e., light source + skin odors + CO_2_) caught more mosquitoes than those traps baited with light source alone [59–61]. Similarly, the present study demonstrated that Suna trap® baited with light and artificial source of CO_2_ caught more *An. arabiensis* than a trap baited with light alone. Furthermore, Suna trap baited with BG lures (i.e., individuals and their combinations), light, and CO_2_ caught mosquitoes three times more than a trap baited with combined light and CO_2_. These findings are consistent with previous studies which show that augmenting light trap with CO_2_ increases catches of outdoor biting mosquitoes than those traps with light alone [62–64]. One of these studies also revealed that traps baited with CO_2_ alone caught more or as equal mosquitoes as traps baited with both CO_2_ and light source [62] or blends [57]. Overall, the current study suggests that the source of CO_2_ alone (i.e., without a source of light) could adequately enhance the attractiveness of designed POHD baited with synthetic blends to mosquitoes.

The existing synthetic odor blends (olfactory cues) and physical cues of warmth were integrated inside the designed POHD, and they were serially evaluated against *An. arabiensis*. This study found that warmth-treated POHD baited with either Mbita or Ifakara blend attracted and killed a significantly higher proportion of *An. arabiensis* than warmth-treated POHD baited with worn nylon socks under the air-tight experimental hut. However, the proportion of mosquitoes attracted to the POHD was halved when evaluated under a setup with free-air movements (screened rectangular box). Such a reduction in the attractiveness of POHD to mosquitoes could be attributed to several factors. Variation in the design of POHD, the addition of an outer cover on the inner bottle, could have compromised the release rate of odor plumes to the surrounding. The bottle inside POHD tested within air-tight experimental hut retained heat and attractiveness to mosquitoes for a relatively longer time than that of POHD within the screened box. Contrastingly, the free-air movement in the screened rectangular box could have diluted the concentration of odor plumes detectable by the mosquitoes. The influence of design in the mosquito collection device on the concentration of odor plumes detectable by mosquitoes has also been reported elsewhere [19]. The findings of this study corroborate with those from previous studies that demonstrated that traps baited with either Mbita blend or Ifakara blend are more attractive than those baited with foot odors in worn socks under open outdoor environment [16, 18, 57]. This study suggests that further investigations are required to evaluate improved POHD baited with synthetic blends against *An. arabiensis* under natural environments.

The textile materials especially nylon strips impregnated with synthetic blends were applied inside fan-powered devices to effectively dispense odor plumes to mosquitoes under both semi- and full-field settings [16, 18, 28, 29, 65, 66]. The current study demonstrated that these nylon strips of Mbita and Ifakara blends can also passively attract substantial numbers of mosquitoes to the designed POHD. But, the short-lived residual attraction of these and other blends when applied on such nylon formats calls for the development of slow-release formulations which would remain effective against mosquitoes for a relatively longer time, thus rendering low-cost POHD. Such slow-release formulations like those of BG lures are indeed urgently needed to enable the sustainable use of POHD baited with synthetic blends (i.e., Mbita or Ifakara blends) and other forms of odor-baited devices for surveillance and control of malaria and possibly other mosquito-borne diseases [34, 57].

While olfactory cues of skin odors and CO_2_ are applied in traps for the long-range attraction of mosquitoes [4, 24, 57, 58, 67], the physical cues especially local convectional currents of warmth determine close-range attraction of mosquitoes [4, 24, 58, 68]. Although warmth enables mosquito to select landing and probing sites on the body of their hosts [4, 24, 58, 69, 70], only a few existing odor-baited devices have incorporated the source of warmth to mimic real humans in attracting mosquitoes [71]. The present study has demonstrated that a bottle of water heated under the sun can passively generate warmth inside the POHD, but such warmth was unable to enhance the attractiveness of Ifakara and Mbita blend baited POHD to mosquitoes. Possibly, the observed similar attractiveness between odor-baited POHD with and without warmth could be associated with the far lower temperature inside the POHD than that of the environment (average 26°C) and human (37°C) for most of the night. For example, the warmth of the bottle containing water decreased with time because the temperatures range from 26.41°C to 32.36°C at 19 : 00 pm to 24 : 00 pm and then cooled down from 26.89°C to 24.98°C from midnight to early morning. This suggests that the proportion of mosquitoes attracted to the POHD in the first three hours was perhaps relatively higher than that collected over other time points in the night. However, this study considered the overall proportion of mosquitoes collected overnight, which in fact is the most realistic way of measuring the efficacy of any mosquito surveillance and/or control tool. This lack of improvement in the attractiveness of Mbita and Ifakara blends when augmented with warmth is consistent with the findings of many similar studies [4, 22, 72]. Other previous studies demonstrated that the surfaces generating higher temperatures (e.g., 44°C to 50°C) prevented mosquitoes from landing and probing [22], but the warmth of 37°C augmented with specific odors and CO_2_ attracted more mosquitoes than individual cues alone [4, 24, 70]. Nevertheless, most of the previous studies concluded that warmth may play a minor role compared to olfactory cues on driving mosquito host seeking behaviors [35, 73, 74].

Although the designed POHD baited with synthetic blends strongly attracted and killed malaria vectors, there are still a number of limitations to be addressed before recommending its mass application in surveillance and control of mosquitoes. The designed POHD was exposed to a 12-year-old colony of semifield reared *An. arabiensis* which may not necessarily represent how the wild population of malaria vectors could respond to POHD in the natural environment. The colonization of *An. arabiensis* may lead to loss of genetic variability under laboratory conditions [75] and small cages under semifield conditions [51]. Contrastingly, our previous work demonstrated that colonizing freely flying population of *An. arabiensis* under the semifield conditions with ambient temperature and relative humidity retains their genetic variability, inbreeding rates, amount of lipids, and body size similar to their founding wild populations [51, 76]. To retain genetic variability in both laboratory and semifield reared mosquitoes (small cages) requires regular replenishment with a collection of founder wild populations. Additionally, the experiments were conducted inside an air-tight experimental hut and screened box within the semifield system which may have omitted some of the ecological factors (e.g., wind speed, direction, and vegetation and host species diversity) found in the natural environments. Therefore, the improved design of POHD baited with synthetic blends needs to be evaluated against freely flying semifield reared mosquitoes and/or wild populations under natural environments.

In conclusion, this study preliminarily demonstrates that the designed POHD baited with synthetic blends and bendiocarb can attract and kill considerable proportions of *An. arabiensis.* The designed POHD baited with synthetic blends such as Mbita and Ifakara blends can effectively attract mosquitoes when blends are delivered in slow-release formulations such as BG lures and augmented with CO_2_. Our future research will, therefore, evaluate the attractiveness of the improved design of POHD baited with a slow-release formulation of synthetic blends and sustainable source of CO_2_ (e.g., CO_2_ mimic compounds such as cyclopentanone) against both semifield and wild populations of malaria vectors.

## Figures and Tables

**Table 1:** Summary of mosquitoes exposed and caught in Suna traps® baited with olfactory or physical cues. The treatments were (a) individual or combined BG lures (LA and NH) augmented with CO_2_ and (b) the source of light alone or combined with CO_2_.

Treatments	Days	Exposed mosquitoes per day	Trap catches
LA	3	200	86
LA + CO_2_	3	200	586
NH	3	200	122
NH + CO_2_	3	200	331
NH + LA	3	200	141
NH + LA + CO_2_	3	200	450
LT	3	200	62
LT + CO_2_	3	200	162

**Table 2:** Multiple comparisons on mosquito catches in Suna traps® baited with BG lures (individually or combined), light source alone, and their augmentation with CO_2_.

Treatments	*z* values	*P* values
LA *∗* NH	2.49	0.12
LA + CO_2_ *∗* LA	15.77	<0.001
LA + CO_2_ *∗* NH	−14.77	<0.001
LA + CO_2_ *∗* NH + CO_2_	−6.9	<0.001
LA + CO_2_ *∗* NH + LA	−14.13	<0.001
LA + CO_2_*∗* NH + LA + CO_2_	2.74	0.006
NH + CO_2_ *∗* LA	11.15	<0.001
NH + CO_2_ *∗* NH	9.44	<0.001
NH + CO_2_ *∗* NH + LA	−8.49	<0.001
NH + LA *∗* LA	3.62	<0.001
NH + LA *∗* NH	1.17	0.84
NH + LA + CO_2_ *∗* NH	12.80	<0.001
NH + LA + CO_2_ *∗* NH + CO_2_	4.25	<0.001
LT + CO_2_ *∗* LT	6.57	<0.001

**Table 3:** Attractiveness of designed passive outdoor host seeking device (POHD) against *Anopheles arabiensis*. The POHD baited with odors augmented with warmth was exposed to these mosquitoes when they are inside (a) air-tight experimental hut and (b) rectangular screened box.

Treatments	Days	Exposed mosquitoes per day	Total mosquito catches	Total dead mosquitoes
(a) Air-tight experimental hut				
Warn socks	3	200	437	317
Control	3	200	437	45
Mbita blend	3	200	489	453
Control	3	200	453	39

(b) Rectangular screened box				
Warn socks	3	100	281	50
Control	3	100	290	9
Mbita blend	3	100	289	87
Control	3	100	289	16
Ifakara blend	3	100	282	97
Control	3	100	288	13

**Table 4:** Multiple comparisons between treatment combinations applied in POHD against *Anopheles arabiensis*. The POHD baited with odors augmented with warmth was exposed to these mosquitoes when they are inside (a) air-tight experimental hut and (b) rectangular screened box.

Treatments	*z* values	*P* values
(a) Air-tight experimental hut		
Socks *∗* Control	17.57	<0.001
Mbita blend *∗* control	20.95	<0.001
Mbita blend *∗* socks	−7.74	<0.001
(b) Rectangular screened box		
Socks *∗* control	6.82	<0.001
Mbita blend *∗* control	10.68	<0.001
Ifakara blend *∗* control	11.72	<0.001
Mbita blend *∗* socks	−3.41	<0.001
Ifakara blend *∗* socks	−4.42	<0.001
Mbita blend *∗* Ifakara blend	−1.10	0.69

## Data Availability

Data generated in this work have been used to support the conclusions made in this study. However, the data will be made freely accessible to the readers upon request from the corresponding author.
